# 2,4,5-Trimethoxyldalbergiquinol promotes osteoblastic differentiation and mineralization via the BMP and Wnt/*β*-catenin pathway

**DOI:** 10.1038/cddis.2015.185

**Published:** 2015-07-16

**Authors:** H-M Yun, K-R Park, T H Quang, H Oh, J T Hong, Y-C Kim, E-C Kim

**Affiliations:** 1 Department of Oral and Maxillofacial Pathology, School of Dentistry and Research Center for Tooth and Periodontal Regeneration (MRC), Kyung Hee University, Seoul 130-701, Republic of Korea; 2 Department of Pharmacy, Institute of Pharmaceutical Research and Development, College of Pharmacy, Wonkwang University, Iksan 570-749, Korea; 3 Department of Pharmacy, College of Pharmacy and Medical Research Center, Chungbuk National University, 12 Gaesin-dong, Heungduk-gu, Cheongju 361-763, Korea

## Abstract

*Dalbergia odorifera* has been traditionally used as a medicine to treat many diseases. However, the role of 2,4,5-trimethoxyldalbergiquinol (TMDQ) isolated and extracted from *D. odorifera* in osteoblast function and the underlying molecular mechanisms remain poorly understood. The aim of this study was to investigate the effects and possible underlying mechanisms of TMDQ on osteoblastic differentiation of primary cultures of mouse osteoblasts as an *in vitro* assay system. TMDQ stimulated osteoblastic differentiation, as assessed by the alkaline phosphatase (ALP) activity, ALP staining, mineralized nodule formation, and the levels of mRNAs encoding the bone differentiation markers, including ALP, bone sialoprotein (BSP), osteopontin, and osteocalcin. TMDQ upregulated the expression of *Bmp2* and *Bmp4* genes, and increased the protein level of phospho-Smad1/5/8. Furthermore, TMDQ treatment showed the increased mRNA expression of Wnt ligands, phosphorylation of GSK3, and the expression of *β*-catenin protein. The TMDQ-induced osteogenic effects were abolished by Wnt inhibitor, Dickkopf-1 (DKK1), and bone morphogenetic protein (BMP) antagonist, noggin. TMDQ-induced runt-related transcription factor 2 (Runx2) expression was attenuatted by noggin and DKK1. These data suggest that TMDQ acts through the activation of BMP, Wnt/*β*-catenin, and Runx2 signaling to promote osteoblast differentiation, and we demonstrate that TMDQ could be a potential agent for the treatment of bone loss-associated diseases such as osteoporosis.

Osteoporosis, the most common bone disease, is a remodeling disease characterized by decreased bone mass, increased bone fragility, and increased risk of fractures.^1^ Potent anticatabolic drugs that include estrogen, bisphosphonates, and RANKL-inhibitor have been the main therapies for osteoporosis.^2^ Furthermore, anti-resorptives have only modest effects on increasing bone mass.^3^ However, the FDA-approved anabolic agent parathyroid hormone has limited use because it is comparatively expensive and difficult to administer.^4^ Thus, it would be most helpful to develop the new and effective anabolic agents that directly stimulate bone formation.

*Dalbergia odorifera*, a traditional herbal medicine in East Asia, has been used to treat various diseases including blood disorders, swelling, ischemia, necrosis, and rheumatic pain.^5^ Previous phytochemical studies reported that the isolated phenolic compounds of *D. odorifera* such as isoflavanones, isoflavans, neoflavonoids, and chalcones exert various beneficial properties, such as anti-oxidant, anti-microbial, and anti-inflammatory effects.^5, 6, 7, 8, 9, 10^ We previously reported that 4,2′,5′-trihydroxy-4′-methoxychalcone, a biologically active chalcone isolated from the heartwood of *D. odorifera*, exhibited protective effects against glutamate-induced oxidative injury in HT22 cells.^10^ In addition, we demonstrated that 4,2′,5′-trihydroxy-4′-methoxychalcone exhibits anti-inflammatory properties by inducing heme oxygenase-1 in murine macrophages.^11^

Although we isolated and identified novel compound 2,4,5-trimethoxyldalbergiquinol (TMDQ) compound from the heartwood of *D. odorifera*, its pharmacological effects in bone-forming cells have not been defined yet. The aim of this work was to investigate the effects and its underlying signal mechanism of TMDQ on osteoblastic differentiation in primary culture of mouse calvarial osteoblasts as an *in vitro* assay system.

## Results

### TMDQ increases osteoblastic differentiation in primary culture of mouse calvarial osteoblasts

**Figure 1:**
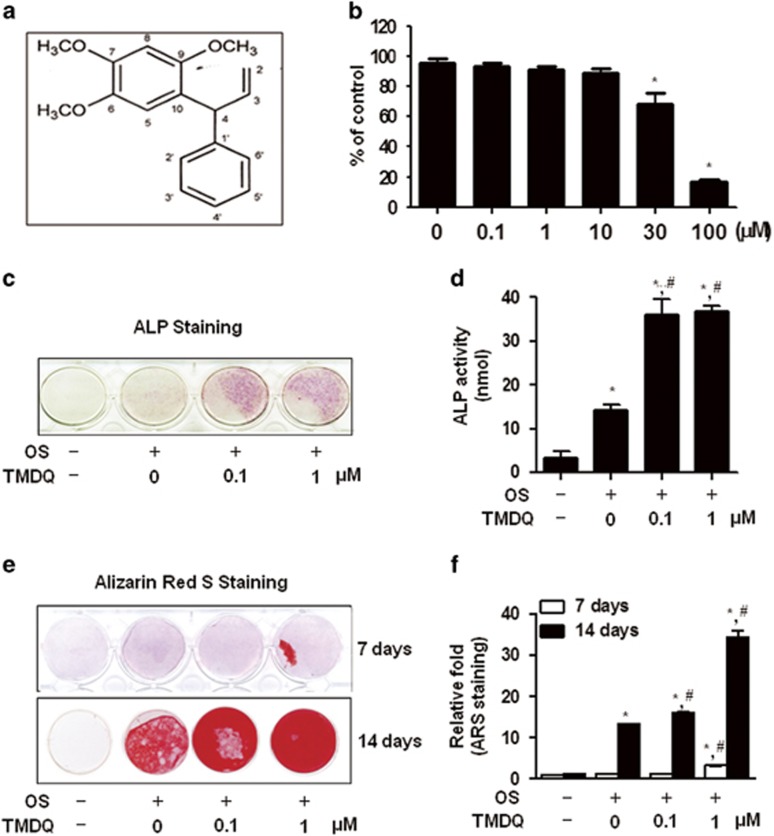
Effects of 2,4,5-trimethoxyldalbergiquinol (TMDQ) on cytotoxicity and osteoblastic differentiation in primary culture of mouse calvarial osteoblasts. (**a**) Chemical structures of TMDQ. (**b**) Cell viability was determined using the MTT assay. (**c**–**f**) Differentiation was assessed by ALP activity (**d**), ALP staining (**c**), and Alizarin red staining (**e**). The intensity of Alizarin red staining was determined by optical density (**f**). Cells were treated with osteogenic supplement medium (OS) containing 50 *μ*g/ml L-ascorbic acid and 10 mM *β*-glycerophosphate with indicated concentration of TMDQ for 1 day (**b**), 5 days (**c** and **d**), 7 and 14 days (**e** and **f**). *P<0.05, *versus* control. ^#^*P*<0.05, *versus* OS. The data presented are representative of three independent experiments

**Figure 2:**
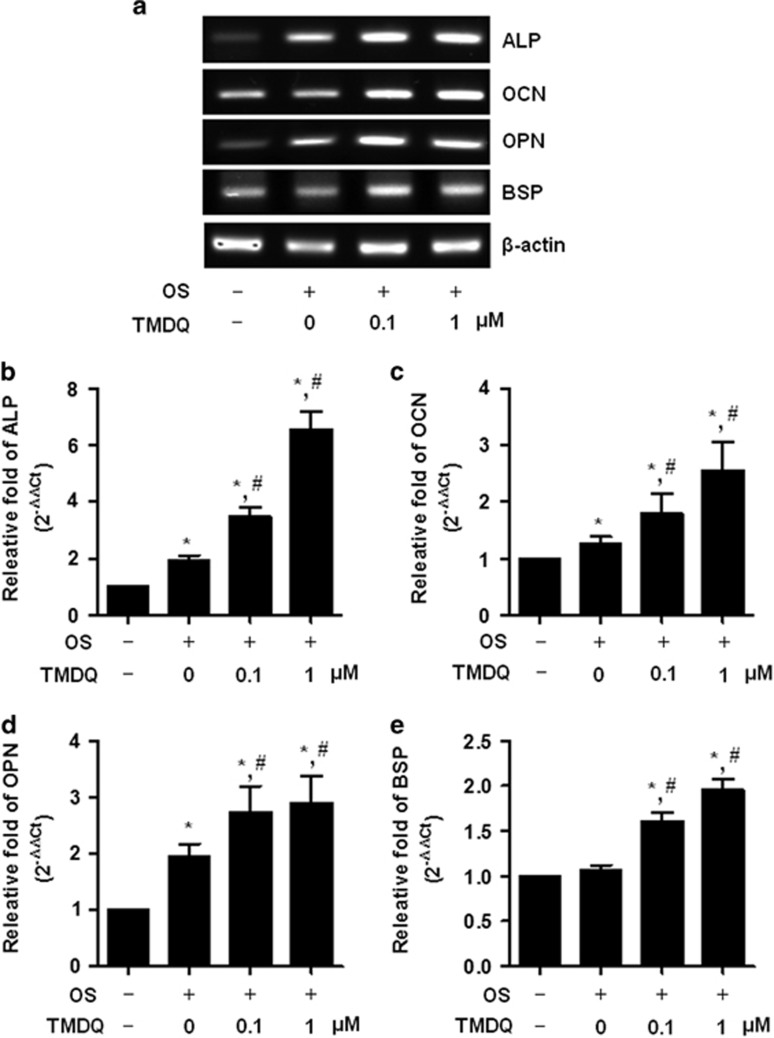
Effects of TMDQ on the mRNA expression of bone matrix proteins in the osteoblasts. (**a**) Cells were cultured in OS with indicated concentration of TMDQ for 7 days. Total RNA was isolated and analyzed by RT-PCR. (**b**–**e**) The level of the target gene expression was determined by real-time PCR, and the values obtained for the target gene expression were normalized to *β*-actin and relatively quantified to the expression in non-stimulated control cells. *P<0.05, *versus* control. ^#^*P*<0.05, *versus* OS. The data presented are representative of three independent experiments

To determine the cytotoxic potential of TMDQ, its effect on viability of the osteoblasts was evaluated. Up to a concentration of 10 μM, no cytotoxic effects could be observed using 3-[4,5-dimethylthiazol-2-yl]-2,5-diphenyltetrazolium bromide (MTT) assay (Figure 1b). To investigate the effects of TMDQ on osteoblastic differentition in the osteoblasts, cells were treated with osteogenic supplement (OS) medium in the presence or absence of TMDQ at non-cytotoxic concentrations (0.1–1 μM). The effect of TMDQ on osteoblastic differentiation was first assessed by measuring the ALP activity as an early phase marker in the osteoblasts. As shown in Figure 1d, TMDQ increased the ALP activity at concentrations ranging from 0.1 to 1 μM. Under the same conditions, the effect of TMDQ on ALP activity was also visualized by ALP staining. As shown in Figure 1c, ALP staining was consistent with ALP activity. The degree of mineralization was determined by Alizarin red staining as a late phase marker. Consistent with the effects on ALP activity and ALP staining, TMDQ increased mineralization by Alizarin red staining (Figure 1e). The quantification of Alizarin red staining showed significant stimulatory effect of TMDQ in a concentration-dependent manner (Figure 2f). As TMDQ significantly increased early and late maker for differentiation in the osteoblasts (Figure 2), we further investigated their effects on mRNA expression for osteoblast-specific genes. As shown in Figure 2, TMDQ also upregulated ALP, osteocalcin (OCN), bone siaophosphoprotein (BSP), and osteopontin (OPN) mRNA expression.

### TMDQ activates bone morphogenetic protein (BMP) and Wnt/β-catenin signaling but not MAPKs signaling

**Figure 3:**
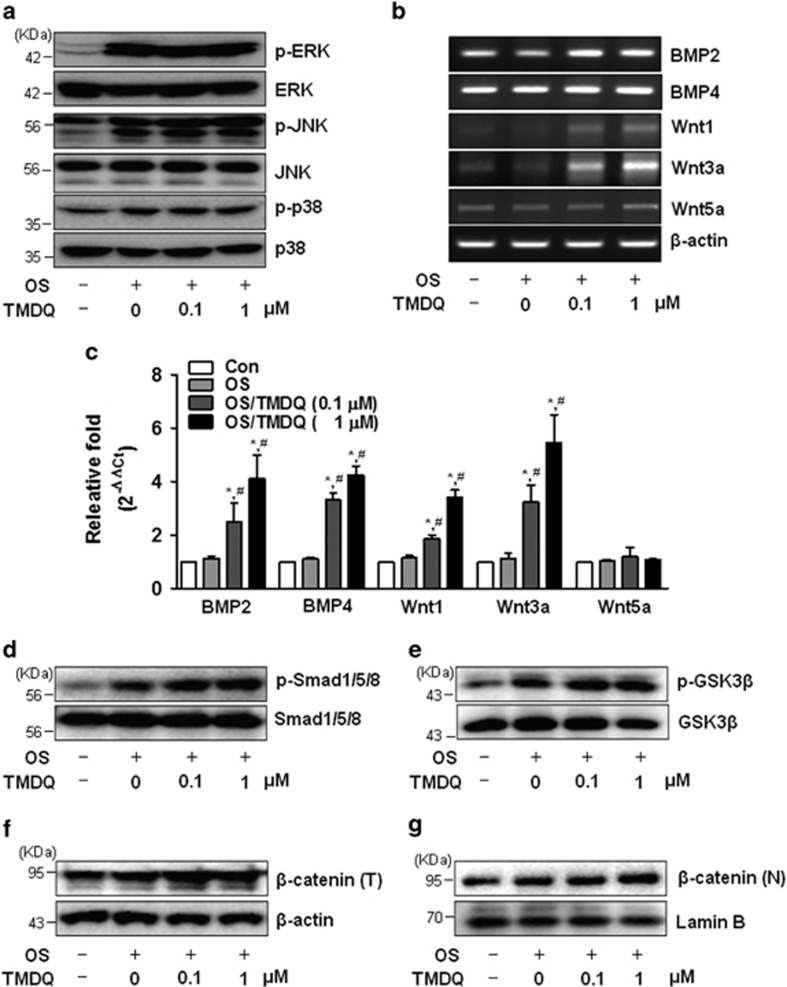
Effects of TMDQ on the activation of MAP kinases, BMP, and Wnt signaling in the osteoblasts. (**a**–**g**) Cells were cultured in OS with indicated concentration of TMDQ for 30 min (**a**) and 2 days (**b**–**g**). mRNA and protein expression levels were assessed by RT-PCR (**b**), real-time PCR (**c**), and western blot analysis (**d**–**g**), respectively. T: total, N: nuclear. The results are representative of three independent experiments

To understand the signal pathways involved in the regulation of cell differentiation by TMDQ, mitogen-activated protein kinase (MAPK), BMPs, and Wnt/β-catenin pathways were analyzed via reverse transcriptase-PCR (RT-PCR), real-time PCR, western blot analysis, and immunofluorescence. As shown in Figure 3a, the treatment of osteoblasts with TMDQ for 30 min did not affect the phosphorylation of ERK1/2, JNK, and p38 MAPK.

**Figure 4:**
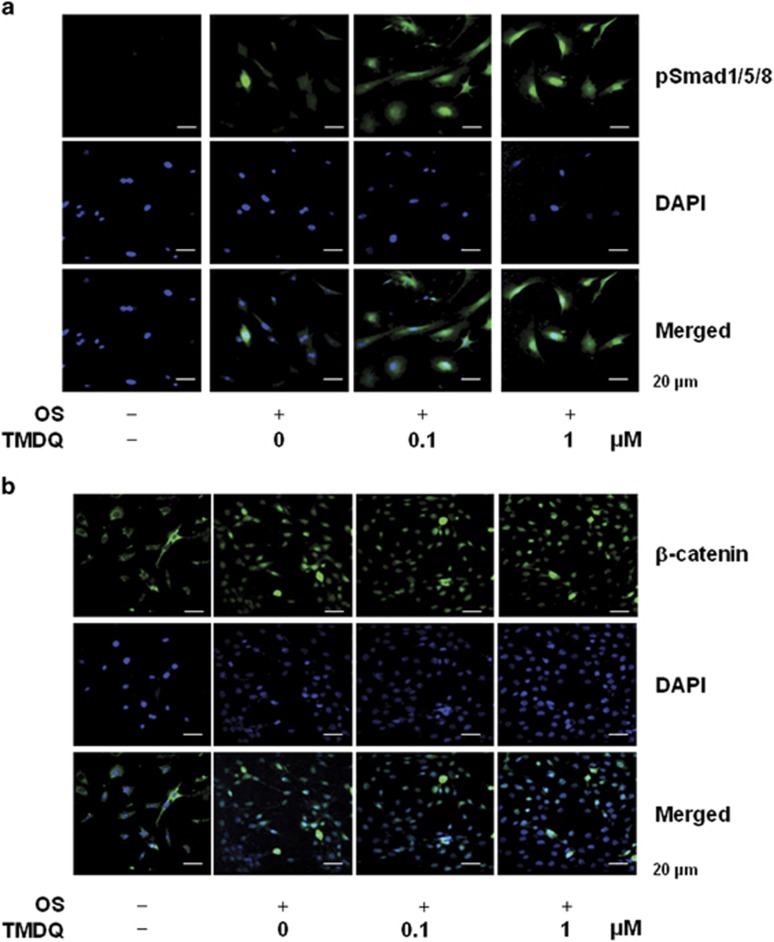
Effects of TMDQ on the nuclear translocation of Smad proteins and *β*-catenin in the osteoblasts. (**a** and **b)** Cells were cultured in OS with indicated concentration of TMDQ for 2 days. (**a**) p-Smad1/5/8 and (**b**) β-catenin were immunostained with rabbit anti-p-Smad1/5/8 or *β*-catenin antibody, followed by Alex488-conjugated secondary antibody (green). And then, the cells were stained with DAPI (blue). The bottom panels show the merged images of the first and second panels. Results are representative of three independent experiments

As BMP pathway has been shown to play an important role in osteoblastic differentiation,^12, 13^ we examined the effect of TMDQ on *Bmp* expression and BMP signaling. We found that TMDQ enhanced *Bmp2* and *Bmp4* mRNA expression as well as the downstream signaling molecules of BMP, and the phosphorylation of Smad1/5/8 in the osteoblasts (Figures 3b–d). To determine whether TMDQ affects Wnt/*β*-catenin signaling, we examined the effects of TMDQ on the mRNA expression of Wnt ligands, the phosphorylation of GSK3, and the expression of *β*-catenin protein. The osteoblasts treated with TMDQ showed the upregulation of Wnt1 and Wnt3a mRNA and phosphorylation of GSK3, but did not affect the expression of Wnt5a mRNA (Figures 3b, c and e). Likewise, the protein levels of total *β*-catenin (T) and nuclear *β*-catenin (N) in the osteoblasts were increased by treatment with TMDQ (Figures 3f and g). To confirm the phosphorylation of Smad1/5/8 and the nuclear translocation of *β*-catenin after the treatment with TMDQ, immunofluorescence staining was carried out. Immunocytochemical observation of TMDQ-treated osteoblasts revealed the rapid nuclear translocation of p-Smad1/5/8 and *β*-catenin (Figure 4).

### TMDQ causes osteoblastic differentiation via the BMPs and Wnt/β-catenin signaling pathway

**Figure 5:**
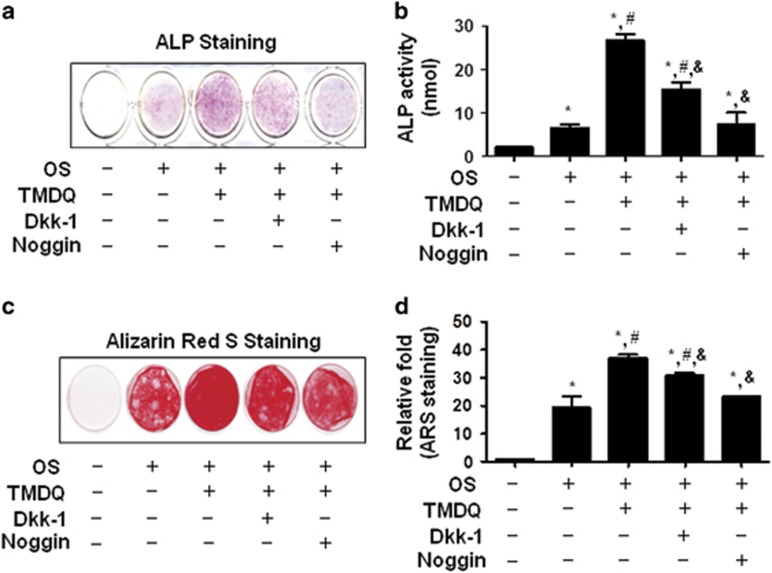
Effects of the inhibition of BMP and Wnt signaling pathway on TMDQ-induced osetoblastic differentiation in the osteoblasts. (**a**–**d**) Cells were pretreated with noggin (10 *μ*g/ml) or DKK1 (0.5 *μ*g/ml) for 1 h, and then cultured in OS with TMDQ for 5 (**a** and **b**) and 14 days (**c** and **d**). ALP activity was measured via ALP staining (**a**) and ALP activity (**b**). Mineralized nodule formation was assessed by Alizarin red staining (**c**), and stains were eluted and measured at 590 nm. The data are represented as relative fold of the control (**d**). *P<0.05, *versus* control. ^#^*P*<0.05, *versus* OS. ^&^*P*<0.05, *versus* TMDQ. Data are representative of three independent experiments

To further investigate whether TMDQ induced osteoblastic differentiation through the activation of the BMP and Wnt/*β*-catenin signaling pathway, cells were pretreated with Wnt inhibitor, Dickkopf-1 (DKK1), and BMP antagonist, noggin, for 1 h before treatment with TMDQ. Results showed that noggin pretreatment drastically inhibited TMDQ-mediated ALP staining, ALP activity, and mineralized nodule formation, whereas DKK1 partially attenuated (Figure 5).

**Figure 6:**
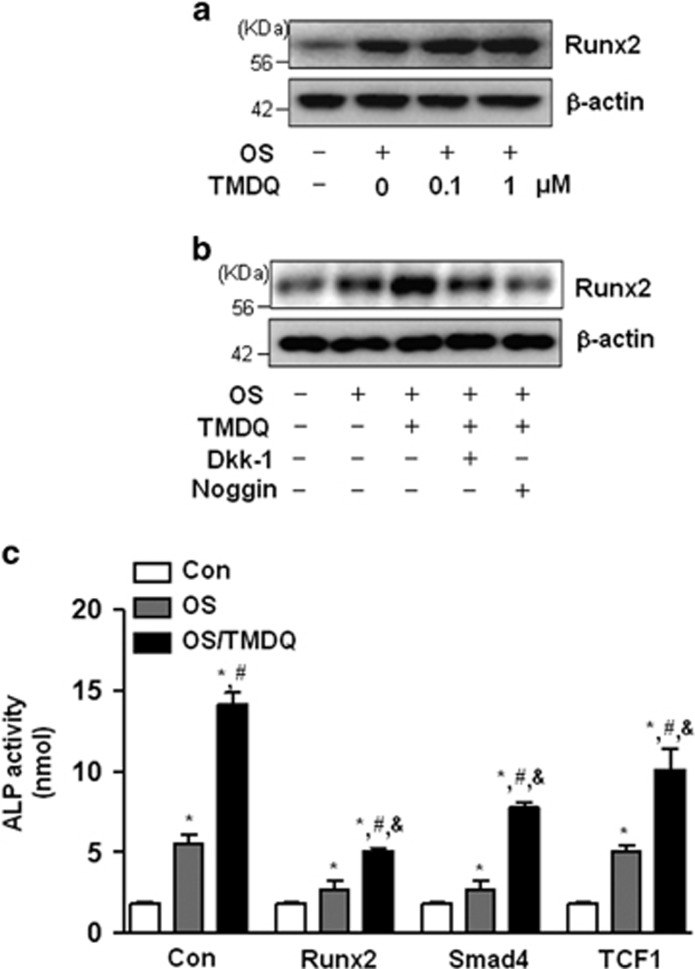
Involvement of Runx2 on TMDQ-induced BMP- and Wnt/*β*-catenin pathways in the osteoblasts. (**a**) Cells were cultured in OS with the indicated concentration of TMDQ for 2 days, and then the expression of Runx2 was determined by western blot analysis. (**b**) Effects of inhibitors for BMP- and Wnt/*β*-catenin on TMDQ-induced Runx2 expression. Cells were pretreated with a BMP inhibitor, noggin (10 μg/ml), or a Wnt/*β*-catenin inhibitor, DKK1 (0.5 *μ*g/ml), for 1 h, and then cultured in OS with TMDQ for 2 days. The expression of Runx2 was assessed by western blot analysis (**b**). (**c**) 24 h after transfection with Runx2, Smad4, and TCF1 siRNAs, cells were cultured in OS with TMDQ for 5 days. ALP activity was measured *P<0.05, *versus* control. ^#^*P*<0.05, *versus* OS. ^&^*P*<0.05, *versus* TMDQ. Data are representative of three independent experiments

To examine the link between the activation of the BMP and Wnt/*β*-catenin pathways, we investigated the effects of TMDQ on *β*-catenin downstream target gene, runt-related transcription factor 2 (Runx2), which is a key transcription factor that has an essential role in osteoblastic differentiation.^14^ The expression of Runx2 was significantly increased by TMDQ after 48 h incubation (Figure 6a). In addition, DKK1 and noggin attenuated TMDQ-stimulated Runx2 protein expression (Figure 6b). To further validate the observation with a genomic approach, the osteoblasts were treated with TMDQ in the presence of Runx2 siRNA to directly downregulate Runx2, as well as its upstream targets, Samd4 siRNA (a final molecule of BMP2-Samd1/5/8 pathway) and TCF1 siRNA (a final molecule of Wnt/beta-catenin pathway). As shown in Figure 6c, Runx2, Smad4, and TCF1 siRNAs attenuated the TMDQ-induced ALP activity. Thus, our pharmacological and genomic approaches suggest that the BMP and Wnt/*β*-catenin pathways are required for TMDQ-mediated osteoblastic differentiation.

## Discussion

Osteoblastic differentiation is a crucial aspect of bone formation and remodeling, which is a process that is severely compromised in osteoporosis. For *in vitro* studies, osteoblasts isolated from calvaria of newborn animals were widely used for osteoblastic differentiation because of relatively pure population.^15^ In the present study, we used primary cultures of mouse calvarial osteoblasts isolated from newborns as an *in vitro* assay system for osteoblastic differentiation. Recently, we reported that 6,4′-dihydroxy-7-methoxyflavanone and 9-hydroxy-6,7-dimethoxydalbergiquinol isolated from *D. odorifera* inhibit osteoclast differentiation and their function, suggesting a potential therapeutic molecule for osteoclastogenic bone diseases.^16, 17^ Our study demonstrated for the first time that TMDQ isolated from *D. odorifera* displayed a stimulating effect on the osteoblasts to induce osteoblastic differentiation.

The formation of new bone involves a complex series of events including the proliferation and differentiation of osteoblasts, and eventually the formation of a mineralized extracellular matrix. Thus, ALP is a phenotypic marker for the early differentiations of osteoblasts, whereas the mineralization of extracellular matrix by Alizarin red staining is a biological marker for terminal differentiation.^18, 19^ Upregulation of ALP, OCN, BSP, and OPN mRNA was markers for the differentiation and maturation of osteoblasts.^18, 19^ The present results showed that TMDQ promoted ALP activity, upregulated the expression of bone matrix proteins (ALP, OCN, OPN, and BSP), and increased mineralized nodule formation in the osteoblasts. These data suggest that the early and late osteoblastic differentiation, and the maturation were stimulated by TMDQ.

BMPs form a unique group of proteins within the transforming growth factor *β* superfamily and have pivotal roles in the regulation of bone formation and remodeling.^20, 21^ BMP signaling is initiated by binding to receptor, propagated by the phosphorylation of Smad1/5/8 complex, and finally translocated into the nucleus to regulate the transcription of target genes.^12, 13^ Several natural or chemical compounds have been reported to induce osteoblastic differentiation by the induction of BMP signaling, such as daidzein,^22^ osthole,^23^ and betulinic acid.^24^ Our data showed that the treatment of osteoblasts with TMDQ enhanced the *Bmp2* and *Bmp4* expression as well as the phosphorylation of Smad1/5/8, which is the central molecule in BMP signaling. In addition, the BMP antagonist, noggin, blocked TMDQ-mediated ALP activity and mineralized nodule formation, indicating that BMP production is required in TMDQ-mediated osteoblastic differentiation. MAP kinases signaling (ERK1/2, p38, and JNK1/2) also represents as an alternative and non-canonical pathway for BMP2 signal transduction to promote osteogenic factors including Runx2.^25, 26^ However, TMDQ did not affect MAP kinases signaling in the osteoblasts. These data suggest that TMDQ promotes osteoblastic differentiation through the BMP/Smad pathway but not the MAPKs pathway.

Wnt/*β*-catenin signaling has critical roles in the bone formation.^27^ *In vitro* and *in vivo* studies showed that the activation of Wnt/*β*-catenin signaling promotes osteoblastic differentiation and mineralization, whereas it inhibits apoptosis in osteoblasts and osteocyte.^19, 24, 28, 29^ The Wnt ligands bind to Frizzled and LRP5/6 receptors, and induce the stabilization of cytoplasmic *β*-catenin by inhibiting GSK3*β*.^30^ Consequently, the *β*-catenin was accumulated in the cytoplasm and translocated into the nucleus to regulate gene expression.^31, 32^ In the present study, we demonstrated that TMDQ significantly increased the expression of canonical Wnt ligands and stabilized the *β*-catenin by inactivating GSK3*β*, allowing the stabilization and nuclear translocation of *β*-catenin. Moreover, the TMDQ-induced osteogenic effects were abolished by Wnt inhibitor, DKK1. These results suggest that TMDQ promotes osteoblastic differentiation and mineralization through the activation of the BMP and Wnt/*β*-catenin signaling pathways.

Runx2 is the main transcription factor required for the activation of osteoblastic differentiation and is crucial for the regulation of genes responsible for the production of bone-specific proteins.^33^ BMP2-mediated Runx2 expression has an important role in the osteoblastic differentiation.^34, 35^ In the present study, the protein level of Runx2 was significantly increased in the TMDQ-treated osteoblasts. In addition, the pharmacological and genomic downregulation for Runx2 attenuated TMDQ-mediated osteoblastic differentiation. It was reported that Wnt/*β*-catenin signaling increases BMP2 expression and vice versa,^36, 37^ as well as osteogenic genes including Runx2 integrate the BMP2 and Wnt/*β*-catenin signaling pathways in the regulation of osteoblastic differentiation.^37, 38, 39, 40, 41^ Based on the above results and the upregulation of Runx2 by TMDQ, Runx2 is activated by the BMP- and Wnt/*β*-catenin-dependent signaling pathways in the osteoblasts, suggesting functional cross talk between BMP2 and Wnt/*β*-catenin siganaling.

In summary, the present study is the first report that TMDQ promotes osteoblastic differentiation through the BMP and Wnt/*β*-catenin signaling pathway to activate Runx2 in primary cultures of mouse calvarial osteoblasts. These findings suggest that TMDQ may be useful in inducing osteogenesis and facilitating bone mineralization for bone diseases such as osteoporosis.

## Materials and Methods

### Isolation and identification of 2,4,5-trimethoxydalbergiquinol

Dried heartwoods of *D. odorifera* (1.65 kg) were extracted with EtOH by untrasonics for 1 h. After concentrated *in vacuo*, the EtOH extract (200 g) was suspended in H_2_O and partitioned with EtOAc to give EtOAc (DOE, 180 g) and aqueous fractions. Fraction DOE was chromatographed over a silica gel column, eluting with acetone in *n*-hexane (5–50%, step-wise) and washing with MeOH to provide seven subfractions (DOE1–6). Subfraction DOE3 was separated by a silica gel column chromatography, eluting with *n*-hexane-CH_2_Cl_2_ (1 : 2) to give five subfractions (DOE31–5). Subfraction DOE34 was further separated by a silica gel column chromatography, eluting with *n*-hexane-EtOAc (4 : 1) to give 2,4,5-trimethoxydalbergiquinol (10 mg). The structure of this compound was elucidated through comparison of the ^1^H- and ^13^C-NMR spectral data with those of the literature.^42^ 4,5-Trimethoxydalbergiquinol: yellow amorphous powder. ^1^H-NMR (CDCl_3_, 400 MHz) *δ* 5.15 (dt, 1.6, 10.4, H-2a), 4.87 (dt, 1.6,16.8, H-2b), 6.26 (ddd, 6.4, 10.4, 17.2, H-3), 5.04 (br d, 6.4, H-4), 6.70 (s, H-5), 6.63 (s, H-8), 7.11–7.24 (H-2′, 3′, 4′, 5′, 6′), 3.69 (s, 6- and 7-OCH_3_), 3.82 (s, 9-OCH_3_). ^13^C-NMR (CD_3_OD, 100 MHz) 111.16 (C-2), 141.98 (C-3), 48.66 (C-4), 115.86 (C-5), 144.76 (C-6), 150.00 (C-7), 99.8 (C-8), 153.1 (C-9), 124.9 (C-10), 144.2 (C-1'), 129.0 (C-2', 6'), 129.5 (C-3′, 5′), 126.9 (C-4'), 56.7 (6-OCH_3_), 56.9 (7-OCH_3_), 57.6 (9-OCH_3_).

### Primary culture of mouse calvarial osteoblasts

Primary osteoblasts were isolated from calvariae of 1-day-old ICR mice after dissected aseptically and treated with 0.2% collagenase-dispase enzyme solution (Sigma-Aldrich, St. Louis, MO, USA). Cells (passage 0) were collected by centrifuge after repeated digestions and cultured in *α*-minimum essential medium without L-ascorbic acid supplemented with 10% fetal bovine serum, penicillin (100 U/ml), and streptomycin (100 μg/ml) at 37 °C in a humidified atmosphere of 5% CO_2_ and 95% air. The cells were detached and reseeded at ~70–80% confluence, and then the cells (passage 1) were used for the experiments reported here. To test the effects of TMDQ on osteoblast differentiation, osteoblastic differentiation was induced by changing OS medium containing 50 *μ*g/ml L-ascorbic acid and 10 mM *β*-glycerophosphate when the cells are ~80% confluent. The recombinant noggin and DKK1 were purchased from Invitrogen (Carlsbad, CA, USA; Catalog Number: PHC1506) and PeproTech (Rocky Hill, NJ, USA; Catalog Number: 120–30), respectively. The medium was replaced every 2 days during the incubation period. TMDQ was dissolved in 100% DMSO and then diluted it (1 : 1000) directly into the medium. Final concentration of 0.1% DMSO was used as the vehicle control. For siRNA transfection, cells were transfected using Lipofectamine RNAiMAX according to the manufacturer's specification (Invitrogen).

### MTT assay

Cell toxicity was measured by an MTT assay to detect NADH-dependent dehydrogenase activity. Fifty microliters of MTT solution (5 mg/ml) in 1 × phosphate-buffered saline (PBS) were directly added to the cells, which was then incubated for 2 h to allow MTT to metabolize to formazan. Absorbance was measured at a wavelength of 540 nm using an ELISA reader (Beckman Coulter, Fullerton, CA, USA).

### ALP activity

ALP activity was measured by spectrophotometry. Cells were homogenized in 0.5 ml distilled water with a sonicator, and centrifuged. The aliquots of cell homogenate were incubated with 15 mM *p*-NPP in 0.1 M glycine-NaOH (pH 10.3) at 37 °C for 30 min. The reaction was stopped by adding 0.25 N NaOH. The absorbance was measured at 405 nm using an ELISA reader (Beckman Coulter).

### ALP staining

Cells were washed with 1 × PBS and then fixed in 4% formaldehyde for 20 min at room temperature. The cells were rinsed with distilled water, and permeabilized by 0.1% Triton X-100. Cells were incubated at 37 °C for 30 min in the Naphthol-AS-BL alkaline solution mixture (Sigma-Aldrich).

### Alizarin red S staining

After 14 days of culture, cells were fixed in 70% ice-cold ethanol for 1 h and rinsed with distilled water. Cells were stained with 40 mM Alizarin Red S (pH 4.2) for 10 min with gentle agitation. The level of Alizarin red S staining was observed under light microscopy. Stains were eluted with 100% DMSO to quantify the amount of Alizarin red staining and measured at 590 nm.

### RT-PCR and quantitative real-time PCR

The total RNA of cells was extracted with Trizol reagent (Life Technologies, Gaithersburg, MD, USA) according to the manufacturer's instructions. One microgram quantity of RNAs isolated from each sample was reverse-transcribed with oligo (dT)_15_ primers with AccuPower RT PreMix (iNtRON Biotechnology, Gyeonggi-do, South Korea). Next, the generated cDNAs were amplified with AccuPower PCR PreMix (Bioneer Corporation, Daejeon, South Korea). The sequences of primers are listed in Table 1. Quantitative real-time PCR was performed using a LightCycler 1.5 Systems (Roche Diagnostics GmbH, Mannheim, Germany). Thermocycling conditions consisted of an initial denaturation of 10 s at 95 °C, followed by 45 cycles of 95 °C for 10 s, 60 °C for 5 s, and 72 °C for 10 s. For the calculation of relative quantification, the 2^−ΔΔ*C*T^ formula was used, where –ΔΔ*C*_T_=(*C*_T,target_–*C*_T,_*β*_-actin_) experimental sample—(*C*_T,target_–*C*_T,_*β*_-actin_) control sample.

### Western blot analysis

Cells were washed twice with ice-cold PBS, and lysed in 20 mM Tris-HCl buffer (pH 7.4) containing a protease inhibitor mixture (0.1 mM PMSF, 5 mg/ml aprotinin, 5 mg/ml pepstatin A, and 1 mg/ml chymostatin). Protein concentration was determined by Bradford reagent (Bio-Rad, Hercules, CA, USA). Equal amounts of lysates (20 *μ*g) resolved on SDS-polyacrylamide gel electrophoresis were transferred to a polyvinylidene fluoride membrane (Millipore, Bedford, MA, USA), and the membrane was blocked with 1 × TBS containing 0.05% Tween-20 and 5% skim milk or 2% BSA for 1 h at room temperature. After blocking, the membranes were incubated overnight at 4 °C with the respective primary antibodies as follows: *β*-catenin (E247; 1 : 1000, #ab32572, Abcam, Cambridge, UK), p-ERK1/2 (1 : 2000, #9101S, Cell Signaling Technology, Beverly, MA, USA), ERK1/2 (1 : 2000, #9102S, Cell Signaling), p-p38 (1 : 1000, #9211S, Cell Signaling), p38 (1 : 1000, #9212S, Cell Signaling), p-JNK (1 : 500, #9251, Cell Signaling), JNK (1 : 1000, #9252S, Cell Signaling), GSK3β (D5C5Z; 1 : 1000, #12456P, Cell Signaling), p-GSK3β (1 : 1000, #9336S, Cell Signaling), Runx2 (O1L7F; 1 : 1000, #12556S, Cell Signaling), p-Smad1/5/8 (D5B10; 1 : 2000, #13820S, Cell Signaling), Smad1/5/8 (N-18; 1 : 1000, #sc-6031-R, Santa Cruz Biotechnology, Santa Cruz, CA, USA), *β*−actin (C4; 1 : 1000, #sc-47778, Santa Cruz Biotechnology), Lamin B (C-20; 1 : 500, #sc-6216, Santa Cruz Biotechnology). The membranes were washed with 1 × PBS and incubated with diluted horseradish peroxidase-conjugated secondary antibodies (1 : 10,000, Jackson ImmunoResearch, West Grove, PA, USA) for 1 h at room temperature. After three washes, the membranes were detected using the enhanced chemiluminescence kit (Millipore).

### Immunocytochemistry

Cells were grown on glass coverslips and incubated with TMDQ for 48 h. Cells were fixed in 10% formalin for 15 min at room temperature. After washing three times in 1 × PBS, the cells were permeabilized with 0.2% Triton X-100 in 1 × PBS for 20 min, washed three times in 1 × PBS, and then blocked with 5% BSA in 1 × PBS for 1 h at room temperature. After then, the cells were incubated with anti-p-Smad1/5/8 (1 : 200, Cell Signaling) and anti- *β*-catenin (1 : 200, Abcam) antibodies for overnight at room temperature, washed three times, and incubated with Alexa-488-conjugated secondary antibodies (1 : 500, Invitrogen) for 2 h at room temperature. The cells was stained with DAPI (Sigma-Aldrich) and washed three times, mounted on glass slides, and viewed on confocal microscopy (Cell Voyager, Yokohama, Japan).

### Statistical analysis

The data were analyzed using the GraphPad Prism version 5 program (GraphPad Software, Inc., San Diego, CA, USA). Data are presented as mean±S.E.M. Satistical significance was performed on the data using one-way analysis of variance and the differences were assessed by the Dunnett's test. A value of *P*<0.05 was considered to be statistically significant.

## Figures and Tables

**Table 1:** RT-PCR primers and conditions

**Genes**	**Primer sequence (5′→3′)**	**Annealing temp (°C)**	**Cycle number**	**Product size (bp)**
*ALP*	F: ACACCTTGACTGTGGTTACTG	58	35	139
	R: CCATATAGGATGGCCGTGAAG			
*OPN*	F: GAGGTGATAGCTTGGCTTATGG	55	30	124
	R: TCCTTAGACTCACCGCTCTT			
*OCN*	F: ACACCATGAGGACCATCTTTC	55	35	148
	R: CGGAGTCTGTTCACTACCTTATT			
*BSP*	F: TGTTTGTAGTGGGCTTCTTCTT	55	35	121
	R: TCCATCTAGTCCCAGCTCATAG			
*BMP2*	F: ACACAGCTGGTCACAGATAAG	58	35	109
	R: CTTCCGCTGTTTGTGTTTGG			
*BMP4*	F: TGCAGACCCTAGTCAACTCT	55	28	110
	R: CACCACCTTGTCATACTCATCC			
*Wnt1*	F: GTTCTGCACGAGTGTCTATGA	55	30	145
	R: GGAGAGATGGATCGCTATGAAC			
*Wnt3a*	F: AGGTAAGCTACTCCCTCAACTA	55	30	115
	R: CTGAAGCACCCTCTCATGTATC			
*Wnt5a*	F: AGCCCAGCTGATTCTTAATACC	55	30	174
	R: GCTCAACTACATGGGACTTTCT			
*Runx2*	F: ACTCTTCTGGAGCCGTTTATG	55	28	103
	R: GTGAATCTGGCCATGTTTGTG			
*β-Actin*	F: AATGTGGCTGAGGACTTTG	55	28	109
	R: GGGACTTCCTGTAACCACTTATT			

## Abbreviations

ALP: alkaline phosphatase
BMP: bone morphogenetic protein
BSP: bone sialoprotein
DKK1: Dickkopf-1
MAPK: mitogen-activated protein kinase
OCN: osteocalcin
Runx2: runt-related transcription factor 2
TMDQ: 2,4,5-trimethoxyldalbergiquinol
OPN: osteopontin
OS: osteogenic supplement
RT-PCR: reverse transcriptase-PCR
